# Virulence genes and antimicrobial resistance pattern in Proteus mirabilis strains isolated from patients attended with urinary infections to Tertiary Hospitals, in Iran

**DOI:** 10.4314/ahs.v21i4.22

**Published:** 2021-12

**Authors:** Azardokht Tabatabaei, Khadijeh Ahmadi, Alireza Namazi Shabestari, Nastaran Khosravi, Ali Badamchi

**Affiliations:** 1 Research Center of Pediatric Infectious Diseases, Rasool Akram Hospital, Iran University of Medical Sciences, Tehran, Iran; 2 Infectious and Tropical Diseases Research Center, Hormozgan Health Institute, Hormozgan University of Medical Sciences, Bandar Abbas, Iran; 3 Department of Geriatric Medicine, School of Medicine, Tehran University of Medical Sciences, Tehran, Iran; 4 Research Center of Pediatric Infectious Diseases, Rasool Akram Hospital, Iran University of Medical Sciences, Tehran, Iran; 5 Children's Medical Center hospital, Tehran University of Medical Sciences, Tehran, Iran

**Keywords:** Antibiotic resistance, Proteus mirabilis, biofilm, virulence factors

## Abstract

**Background:**

Proteus mirabilis is a frequent reason for catheter-associated urinary tract infections (UTIs). The aim of this study was to identify virulence genes and antimicrobial resistance patterns in P. mirabilis strains isolated from patients who attended a tertiary hospital in Iran.

**Methods:**

In this study, 100 P. mirabilis strains from urine samples were isolated. These isolated strains were identified by biochemical and PCR-based tests, and their antibiotic resistance was profiled through a standard procedure using 14 antibiotics. PCR assays were used to detect virulence-related genes in P. mirabilis strains. The biofilm formation of each P. mirabilis strain was examined.

**Results:**

Of the 100 P. mirabilis isolates, 16 (16%) were multidrug-resistant. High resistance was observed against cotrimoxazole (97%), nalidixic acid (93%), cefotaxime (77%), and amoxicillin (62%). Sixty of the 100 isolates showed resistance against extended-spectrum cephalosporins. The prevalence rates of the genes related to the virulence factors in this study were mrpH (100%), ucaA (91%), hpmA (94%), zapA (95%), ptaA (100%), ureG (100%), pmfA (100%), fliC (97%), and mrpA (90%) using PCR method. Strong biofilm formation was observed in 20% (5/25) of the strains isolated from non-catheterized samples and 80% (20/25) of strains isolated from catheterized samples.

**Conclusions:**

Resistance to antibiotics and the prevalence of pathogenicity genes are high in Proteus mirabilis strains iolated from UTIs.

## Background

Indwelling urinary catheters are the most commonly used medical devices and are employed in a wide range of bladder management regimens in hospitals, community care settings, and nursing homes^1^. It has been estimated that millions of urethral catheters are used each year, with many applied for long-term bladder control in public or nursing home settings^2^.

P. mirabilis is investigated as a frequent reason for catheter-associated urinary tract infections (UTIs), which can be caused by urolithiasis, and the development of bladder or kidney stones arises from the alkalinization of urine from urease-catalyzed urea hydrolysis^3^. The bacterium is Gram-negative and rod-shaped and is recognized by its swarming activity.

Swimming motility may facilitate contact with uroepithelial cells, thereby promoting internalization and cytotoxicity^4^. Swarm cells, in particular, have been postulated to contribute to host cell invasion because these differentiated cells can invade urothelial cells faster and more prominently than vegetative cells^5^. Swimming motility is also thought to contribute to dissemination within the urinary tract, in particular regarding the ascension from the bladder to the kidneys and spread between kidneys. Flagella clearly contribute to *P. mirabilis* pathogenesis^6^.

Three potential toxins have been characterized for their important role in virulence. These are hemolysin, Proteus toxic agglutinin (pta), and ZapA metalloprotease. Hemolysin could play a role in the spread of infection into the kidneys and the initiation of acute pyelonephritis. pta is an auto-transporter that performs serine protease activity on the surface of bacteria^7^, ^8^. The pta protein contributes to the colonization of the bladder and kidney. In vitro and in vivo UTI studies have demonstrated the additive effects of hpmA and pta, particularly with respect to cystitis and possibly interstitial nephritis^9^–^11^. ZapA Metalloprotease is capable of cleaving IgA, IgG, antimicrobial peptides hBD1 and LL-37, complement proteins C1q and C3, fibronectin, actin, collagen, laminin, casein, and gelatin. Zap protease and hemolysin may cause swarm cells to be more cytotoxic to the host urothelium^5^.

Bacteria utilize quorum sensing to control biofilm, toxin, exopolysaccharide, virulence factor production, and motility, all of which are necessary for the effective foundation pathogenic relation with eukaryotic hosts^11^. *P. mirbilis* fimbriae contribute to infection in the kidney and bladder, although the receptors involved have not been recognized yet. The attachment of fimbriae to renal cells to initiate pyelonephritis and the critical role of fimbriae in cystitis have been demonstrated in previous studies^12^. Mannose-resistant Proteus-like fimbriae (MR/P) are expressed in the urinary tract and contribute to virulence. Direct observations of *P. mirabilis* in the bladder, urine, and kidneys of mice revealed MR/P fimbriation in all parts of the urinary tract^13^. However, up to 85% of bacteria do not express MR/P in the kidneys^14^. Phase variation of the mrp promoter orientation may contribute to the evasion of host defences.

The formation of biofilms on catheter surfaces, including urinary catheters, is considered a notorious problem. The formation of biofilm depends on MR/P fimbriae. The formation of biofilm in catheters and urinary tissue is associated with *P. mirabilis* catheterization. Crystalline biofilms are formed by depositing struvite and apatite minerals among the colonized surfaces in the presence of urine. The urease activity of bacteria increases the local pH, eventually leading to the deposition of minerals^15^. Crystalline biofilms can obstruct catheters, and, therefore, *P. mirabilis* is especially problematic for patients with indwelling urinary catheters.

*P. mirabilis* is isolated between 1–10% of all UTIs, without considering the geographic location of the study, the types of samples collected. In recent studies, this species was found in 5–20% of cases and had a mortality rate of as high as 50% in geriatric patients^7^, ^16^.

*P. mirabilis* has a broad range of virulence factors, each of which plays a vital role in UTIs. These factors are associated with the relationship between bacteria and surfaces, invasion, damage to host tissues escaping from the host's immune system, and iron absorption^17^.

There is a lack of research considering the epidemiology, and prevalence of serogroups, the frequency of virulence factors, and the characteristics of antibiotic resistance regarding *P. mirabilis* in Iran. This study was designed to characterize virulence genes and antimicrobial resistance patterns in *P. mirabilis* isolated from patients with urinary infections who were admitted to tertiary hospitals in Iran.

## Methods

In our study, a total of 100 isolates of *P.mirabilis* causing UTIs were isolated from patients and outpatients in hospitals affiliated to University of Medical Sciences of Tehran, Iran from August 2016 to August 2018. The selection of the subjects in this study was in accordance with CDC guideline. The recruited patients met the following criteria: temperature more than 38°C (fever) need to severe urinary excretion, with frequent urination, Dysuria, incomplete bladder emptying, Supra-pubic and flank pain, presence of leukocytes or blood in the urine and finally, a positive culture with more than 105≥CFU/ml colonies. Information on patients including the types of UTIs, relapses, age; sex and etc. were collected with the patient's consent.

### Identification and preservation of *P. mirabilis* strains

Identification of the strains was performed using the API tests: API 20E/ID32E (BioMérieux), according to the manufacturers' recommendations. Strains were stored in a brain heart infusion with 20 % glycerol at -70 °C.

### Antimicrobial susceptibility testing

The antimicrobial susceptibility testing was performed using the disk diffusion method to amoxicillin with clavulonic acid, piperacillin with tazobactam, cefotaxime, cetriaxone, cefepime, amikacin, gentamicin, ciprofloxacin, nalidicxic acid, trimethoprim-sulfamethoxazole(SXT), imipenem, and meropenem according to the Clinical and Laboratory Standards Institute (CLSI 2017) guidance 18, 19.

### Evaluation of biofilm Formation

The biofilm formation of the *P. mirabilis* strains was examined using the modified method described by Kwiecinska-Piróg et al. A 0.1% (TTC) 2, 3, 5-Triphenyl-tetrazolium chloride solution with modifications was applied. After a 24-hour incubation at 37 ºC, planktonic, and non-adsorbed cells were washed from the wells. The wells were washed three times with 600 µL sterile distilled water (DDW). Then, 100 µL of tryptic soy medium (TSB, Becton Dickinson) and 100 µL of sterile 0.1% TTC solution were added to each well and incubated for two hours at 37 ºC temperature. The contents of the wells were removed and washed again with DDW. The formazan was suspended in 200 µL methanol and added to each well. The contents of the wells were moved to sterile microtiter plates. The absorbance was counted using a BIO-TEK spectrophotometer at 470 nm. The results were interpreted in accordance with the criteria described in the previous publication^20^,^21^.

### Molecular detection of virulence genes

Genomic DNA was extracted by DNA extraction kit (Roche, Switzerland) and stored at -20 °C. PCR approach was used to detect the presence of virulence genes including (mrpH, ucaA, hpmA, zapA, ptaA, ureG, pmfA, fliC, mrpA). The primers of this study were specifically designed and synthesized by Gene Fannavaran Company (Iran). The primer sequences, their annealing temperatures and product sizes are given in Table 1. The PCR was performed in Eppendorf Thermal Cyclers (Eppendorf, USA) 22.

**Table 1:** list of primers which were used in this study

	primer sequence (5 3)	Target genes	Size(bp)	Annealing Tem	Reference
1	F: TTC TTA CTG ATA AGA CAT TG R: ATT TCA GGA AAC AAA AGA TG	*mrpA*	512	56	This study
2	F:CTGCGGCTTTAGTATTTGGT R:TAACGGCTTGGAATTCACCT	*pmfA*	504	47	This study
3	F:CATGCCATGAAAAGAAAAGTTATAGC R:CCCAAGCTTCTCATAGGCAATGGTGTAAT	*ucaA*	505	56	This study
4	F:AGAATATAATCAACCACTGCGTA R:CATTTTGGCTGTATCCGCTTC	*ureG*	514	47	This study
5	F:CAATTTCAGCACCTAATAACCC R:TGCTTAATCAAGGAGCCGAT	*ptaA*	636	47	This study
6	F:ATAGTCACGCCAAATAACGAA R:TATTTCCACGAGTAGAACCAG	*hmpA*	971	47	This study
7	F:TATCGCAGAACTGATCACTCG R:ATCTGGCTCTTTGTTAGCTTG	*zapA*	541	47	This study
8	F:CATGCCATGGCGATGGCACAAGTCATTAAT R:CCGCTCGAGACGTAACAGAGACAGAACA	*fliC*	1100	55	This study
9	F:CATGCCATGGCCATGTTTATATTTAAACGA TT R:CCCAAGCTTAGGCATGGTTAAAATAATTG	*mrpH*	828	55	This study

### Statistical analysis

Data were analyzed using statistical analysis software, SPSS version 24.0 (IBM, Chicago, USA). Categorical data were analyzed using chi-square or Fisher's exact tests. P-value <0.05 was considered significant.

## Results

### Isolation of *P. mirabilis*

A total of 100 specimens representing patients clinically were diagnosed as UTI patients distributed as 40 Isolates from males and 60 Isolates from females. 35 (35%) strains were isolated from urine collected from catheterized, while 65 (65%) strains were isolated from the urine of non-catheterized patients.

### Antimicrobial Susceptibility Profile

**Fig 1:**
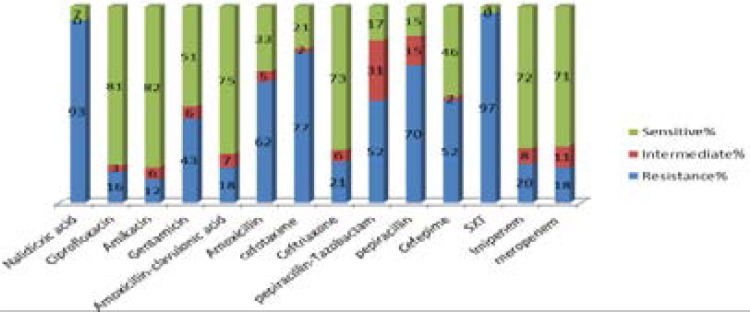
The susceptibly pattern of 100 *Proteus mirabilis* isolates to 14 antimicrobial agents.

The results of the antibiotic susceptibility testing are shown in Figure 1. Of the 100 P. mirabilis strains isolated from UTI, 3 (3%) were susceptible to all antimicrobials tested, while 16 (16%) were observed to be unsusceptible to more than three antimicrobial families and were identified as multidrug-resistant (MDR). Twenty isolates were resistant to imipenem and 18 isolates were resistant to meropenem. 77 (77%) isolates demonstrated resistance to the extended spectrum β-lactam antibiotics in the disk diffusion test. Nalidixic acid SXT had the highest resistance. The antimicrobial susceptibility profile of the P. mirabilis isolates revealed that 72% and 71% were susceptible to imipenem, meropenem, 82% to amikacin, 81% to ciprofloxacin, and 73% to ceftriaxone. Antimicrobial resistance was observed against the SXT (97%), nalidixic acid (93%) and amoxicillin (62%) antibiotics.

### Circulation of Virulence Genes in *P. mirabilis* isolated from UTI

**Fig 2:**
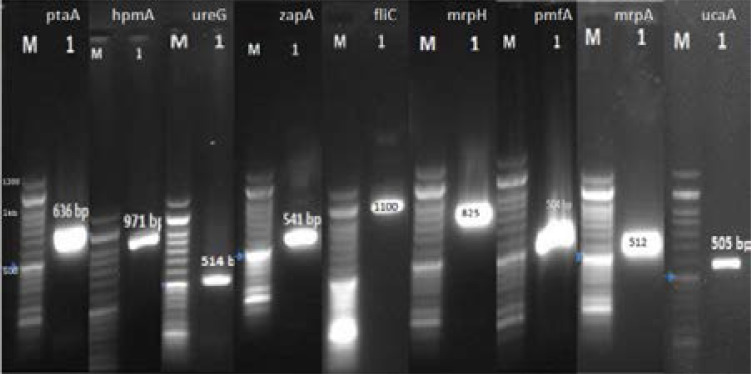
An electrophoresed gel showing PCR products. The left most lane represents a DNA ladder with fragments at 100bp intervals.

Among the nine virulent genes, mrpA was detected at a ratio of 90% (90/100), while mrpH, ptaA, ureG and pmfA exhibited a similar percentage; 100% (100/100) was observed in *P. mirabilis*. Moreover, among the nine virulent genes, ucaA was detected at a ratio of 91% (91/100), while zapA and fliC exhibited similar percentages; 95% of zapA, and 97% of fliC were observed in *P. mirabilis* (Fig 2).

### Comparison of the urine-derived *P. mirabilis* strains; the ability to form a biofilm with respect to catheterization of the patients

The potency to form biofilm by *P. mirabilis* strains isolated from non-catheterized and catheterized patients showed significant differences. A higher percentage (83.3%) of the strains in this case was classified as weak biofilm producers among non-catheters. A higher percentage (80%) of the strains in this case was classified as strong biofilm producers among catheters patients (Table 2).

**Table 2:** Comparison of the urine-derived *P. mirabilis* strains ability to form biofilm with respect to catheterization of the patients

Biofilm		Lack	Weak	Moderate	Strong	
Patients	N (%)	50	12	13	25	P value
Catheterized	35(35%)	5(10%)	2(16.7%)	8(61.5%)	20(80%)	0.00001
Non- Catheterized	65(65%)	45(90%)	10(83.3%)	5(38.5%)	5(20%)	

## Discussions

The emergence of MDR strains that are resistant to most of the tested antimicrobials agents might be because of the use of easily available prescription and non-prescription drugs before the urine culture results were obtained. The widespread use (and often misuse) of antimicrobial drugs has led to a general rise in the emergence of resistant bacteria.

The results also showed that 62 (62%) and 70 (70%) isolates were resistant to amoxicillin and piperacillin, respectively. These surveys are consistent with studies that showed that Proteus isolates were unsusceptible to ampicillin and piperacillin and that reported that ampicillin has no effect on any of the isolates of UTIs 22.

A major virulence factor of these bacteria is their ability to create a biofilm. Biofilm protects bacteria from the host's immune system response and restricts the penetration of antibiotics and antibodies 23.

The typical effects of the biofilm-trapped bacteria are an almost-1000-fold increased resistance to most antimicrobials when compared to planktonic bacteria. The biofilm formed on the abiotic surfaces is a major cause of 65% of nosocomial infections 20.

*P. mirabilis* showed the potential to create biofilm in various environments, including on abiotic (catheter) and biological surfaces 24. It might cause urine obstruction in the bladder, bacteriuria recurrent, fever, sepsis, and shock. 25

In the present study, 90% of strains had the mrpA gene, inclding 70% in cystitis and 30% in pyelonephritis. Sosa et al. studied clinical and non-clinical strains and found that several virulence factors (e.g., swarming, urease) are associated with the uropathogenic *P. mirabilis*. Hemolysin production and various instances of fimbrial gene expression were analyzed; the data showed that all the strains have mrpA, pmfA, and ucaA genes 26.

In another study on urinary *P. mirabilis* strains in which virulence factors such as urease, protease, hemolysin, and the ability of swarming/span>were evaluated and measured, all studied strains had ureC and zapA genes^27^. The prevalence rates of the genes related to the virulence factors based on the multiplex PCR method were ureA (96.7%), ureC (100%), hpmA (100%), zapA (100%), and flaA (86.7%) 27. The majority of the isolated strains in the current study contained fliC, zapA, hpmA, and ucaA genes; similar results were obtained in other studies 28.

The zapA and mrpA genes are mainly important for the adherence were identified in 95% and 90% isolates, respectively. However, in the study by Holling et al., the frequencies of both genes in *P. mirabilis* was 73.3%, which contrasts with other previous studies, which reported a frequency of 30% 29, 30. In our study, the most important gene was mrpA per its role in several virulence factors 30. The genes involved in biofilm formation, such as pmfA, ucaA, mrpH, mrpA, and fliC, were found in the majority of the strains, possibly resulting in high-intensity biofilm formation and thus increasing antibiotic resistance among the strains 20.

*P. mirabilis* with controlling antibodies and antimicrobial peptides can lead to UTIs. To do this, *P. mirabilis* encodes a protease that is capable of mediating the degradation of the β-defensin-1 and LL-37 that are present in the urinary tract 20, 30. The ZapA-mediated degradation of β-defensin-1 and LL-37 decreases their antimicrobial activity 31.

The formation of biofilm, evaluation of hemagglutination, and measurement of virulence markers were studied in the urinary *P. mirabilis* strains of patients with UTIs in Italy (2006). The results showed that all the strains contained mrpA and mrpH genes 32.

Biofilms, which are adherent microbial communities, are a notorious problem on catheter surfaces (including urinary catheters) and contribute to disease. Because catheterization is a major risk factor for *P. mirabilis* UTIs, biofilms within catheters and urinary tissues must be considered. MrpA is the main structural subunit of MR/P, and its expression is increased when oxygen is limited, which is logical for a virulence factor, given the reduced oxygen availability in the bladder 11.

Experiments conducted by Tsai et al.^25^ suggest that MR/P fimbriae dictate the localization of bacteria in the bladder and contribute to biofilm formation, a process that is essential for the establishment of catheter-associated UTIs. MR/K fimbriae can cause the adhesion of bacteria to catheter surfaces and the permanence of catheter-related bacteriuria.

## Conclusion

The *P. mirabilis* strains isolated in the current study are accompanied by several virulence factors, including adherence factors, hemolysin, urease, and swarming activity. The presence of important virulence factors was further validated using a PCR approach.

## Abbreviations

UTIs: Urinary Tract Infections; PCR: Polymerase Chain Reaction; ICU: Intensive Care Unit; CLSI: Clinical and Laboratory Standards Institute

## Declarations

### Availability of data and materials

The datasets used and/or analyzed during the current study are available from the corresponding author on reasonable request.
